# ZFP36 Family Proteins as Critical Regulators of Inflammation, Immune Cell Development, and Antiviral Responses

**DOI:** 10.3390/vaccines14080656

**Published:** 2026-07-27

**Authors:** Malabika Bhowmik, Tooba Momin, Neelu Thakur, Neeraj Singh, Mrigendra Rajput

**Affiliations:** 1 Richard A. Gillespie College of Veterinary Medicine, Lincoln Memorial University, Harrogate, TN 37752, USA; malabika.bhowmik@lmunet.edu (M.B.); t.momin@lmunet.edu (T.M.); neelu.thakur@lmunet.edu (N.T.); 2 Department of Physiological Sciences, College of Veterinary Medicine, Oklahoma State University, Stillwater, OK 74078, USA; neeraj.singh@okstate.edu

**Keywords:** RNA-binding proteins, tristetraprolin (TTP), ZFP36L1, ZFP36L2, antiviral proteins, immune modulation

## Abstract

The innate immune system provides the first line of defense against invading pathogens and relies on tightly regulated mechanisms to initiate and resolve inflammatory responses. In addition to transcriptional control, post-transcriptional regulation of messenger RNA (mRNA) plays a critical role in determining the magnitude and duration of immune responses. Among those key regulators, the ZFP36 family of CCCH-type zinc-finger RNA-binding proteins, including ZFP36, ZFP36L1, and ZFP36L2, plays a central role in controlling the stability and translation of inflammatory, immune-related, and viral RNAs. This review focuses on the ZFP36 family of RNA-binding proteins and highlights their emerging roles in inflammation, immune cell development and differentiation, and antiviral immunity, with a particular focus on ZFP36L1 and ZFP36L2. Recent evidence has identified ZFP36L1 as a broad-spectrum antiviral factor that restricts multiple RNA viruses through distinct molecular mechanisms. A detailed understanding of the molecular mechanisms underlying ZFP36L1- and ZFP36L2-mediated antiviral activity and immune regulation will facilitate rational development of host-directed antiviral therapeutics and their strategic integration with vaccination strategies to limit viral replication, shedding, and transmission, thereby improving the control of emerging and re-emerging viral diseases.

## 1. Introduction

The innate immune response represents the first line of host defense against invading pathogens [1]. It responds rapidly, provides immediate protection against invading pathogens while directing the subsequent adaptive immune response [2]. Importantly, the innate immune system also plays a central role in initiating and shaping vaccine-induced immunity, as vaccines rely on innate immune pathways to activate robust adaptive immune responses. The innate immune response is coordinated through multiple interconnected mechanisms, including detection of pathogen-associated and damage-associated molecular patterns, interaction with pattern-recognition receptors (PRRs) such as Toll-like receptors (TLRs) or retinoic acid-inducible gene-I-like receptors (RIG-I–like receptors) and activation of the complement system and downstream signaling pathways [1,3,4]. Together, these pathways trigger intracellular signaling cascades that induce cytokines, chemokines, and interferon-stimulated genes (ISGs) [5,6,7]. These mediators act to suppress pathogen replication and coordinate responses to tissue injury by recruiting and activating immune cells such as neutrophils, macrophages, and dendritic cells, thereby orchestrating an effective immune response [8]. These innate signaling pathways are equally critical for vaccine adjuvant activity and the development of protective immune memory.

Like adaptive immunity, the innate immune response is tightly regulated to ensure effective activation followed by timely resolution. Excessive or prolonged innate immune signaling can lead to tissue damage, chronic inflammation, or autoimmune diseases [9]. Several regulatory mechanisms control the initiation and termination of innate immune responses. Among these, post-transcriptional regulation of cytokine, chemokine, and interferon-stimulated gene (ISG) expression plays a critical role [10]. Control of mRNA stability, translation, and subcellular localization is a key mechanism in post-transcriptional regulation that determines the timing, strength, and duration of inflammatory responses.

One important feature of post-transcriptional regulation is that many inflammatory and stress-responsive mRNAs are inherently unstable due to enrichment of cis-regulatory sequences within their 3′ untranslated regions (3′ UTRs), particularly AU-rich elements (AREs) [10]. AREs serve as binding platforms for trans-acting RNA-binding proteins (RBPs), which can promote rapid mRNA degradation or repress translation. This enables host cells to rapidly initiate and terminate cytokine production, supporting effective responses to infection while limiting prolonged inflammation [11,12].

Members of the ZFP36 family of RNA-binding proteins play a central role in regulating this transcript triage process [13]. These proteins bind ARE-containing mRNAs in a sequence-specific manner and direct them either toward mRNA decay pathways or toward translational repression. These proteins therefore play an important role in regulating innate immunity by acting as intermediaries between signaling pathways and protein synthesis and by determining mRNA fate [13,14].

Although previous reviews have primarily focused on the role of the ZFP36 family proteins in inflammation, immune regulation and immune cell development and differentiation [15,16], accumulating evidence has demonstrated that the family members ZFP36L1 (formerly known as butyrate response factor 1 [BRF1]) and ZFP36L2 (formerly known as butyrate response factor 2 [BRF2]) also function as broad-spectrum antiviral restriction factors against multiple RNA viruses [17,18,19].

These discoveries substantially broaden the biological significance of the ZFP36 family beyond the regulation of inflammatory mRNA turnover. Accordingly, this review integrates current knowledge of the emerging antiviral mechanisms of ZFP36L1 and ZFP36L2, discusses the potential phosphorylation-dependent regulation of ZFP36L1 and ZFP36L2 and how phosphorylation may balance their antiviral and immunomodulatory activities.

## 2. Methods

This review used a narrative approach to provide a comprehensive summary of current knowledge on the ZFP36 family of RNA-binding proteins, with particular emphasis on their roles in immune regulation. Specifically, this review focuses on the post-transcriptional regulatory functions of ZFP36 proteins in controlling inflammatory responses, as well as immune cell development and differentiation. The initial part of this review is organized the molecular pathways underlying ZFP36 family function, whereas the later sections focus on their emerging antiviral activities. Notably, the antiviral role of ZFP36L1 has only recently been demonstrated, with the first studies reporting its antiviral activity emerging in recent years.

Relevant literature was identified through searches of multiple scientific databases, including PubMed and Google Scholar, as well as relevant theses, dissertations, and review articles. Literature research was conducted between January 2026 and March 2026, without restrictions on publication date, to include both recent findings and foundational studies. Studies were selected based on their relevance to ZFP36-mediated post-transcriptional regulation, immune responses, and antiviral mechanisms. Although no formal language restrictions were applied, only studies available in English were included.

Inclusion criteria comprised peer-reviewed research articles, reviews, and relevant reports addressing the molecular functions of ZFP36 family proteins, their role in immune regulation, and their antiviral activity.

As a narrative review, this study has inherent limitations. It does not employ systematic quantitative synthesis or meta-analysis, which may introduce potential selection bias. Nevertheless, the use of multiple reputable databases and a broad range of scientific literature provides a comprehensive overview of current knowledge and emerging insights into the roles of the ZFP36 family proteins in immune regulation and antiviral defense.

## 3. Overview of Post-Transcriptional Regulation in Immunity

The immune system relies on a multi-layered regulatory network to mount an effective defense while avoiding harmful overreactions. Transcriptional activation of immune genes establishes the primary level of regulation, whereas post-transcriptional control of mRNA constitutes a critical secondary regulatory layer that modulates the magnitude and duration of immune responses [20]. Post-transcriptional mechanisms regulate the stability, localization, and translational efficiency of mRNAs, allowing cells to rapidly and reversibly adapt in response to environmental and physiological changes [13]. This process is essential during inflammatory and antiviral responses, where the precise regulation of cytokine production can determine the distinction between protection and pathology.

Key mediators of post-transcriptional gene regulation include RNA-binding proteins (RBPs), microRNAs, long non-coding RNAs (lncRNAs), circular RNAs (circRNAs) and host machinery that spatially regulates mRNAs through localization and subcellular compartmentalization [11,21,22,23,24,25,26,27]. RNA-binding proteins (RBPs) regulate immune gene expression by binding to cis-regulatory elements, most notably AU-rich elements (AREs), within target mRNAs to control their stability, translation, and decay, thereby enabling the rapid induction and timely termination of cytokine responses [11]. AREs are conserved sequence motifs enriched in the 3′ untranslated regions (UTRs) of many immune-related transcripts, particularly those encoding cytokines, chemokines, and interferons [28,29]. Through cooperative or competitive interactions with microRNAs at these elements, RBPs can direct mRNAs toward degradation or translational repression, thereby precisely regulating protein output and preventing excessive inflammation [30].

Unlike microRNAs that directly bind target mRNAs, lncRNAs regulate immune gene expression indirectly by guiding regulatory proteins or RNAs to specific targets [22]. Similarly, circRNAs, which are more stable than lncRNAs, regulate immune responses by sequestering microRNAs or RNA-binding proteins on the circRNA molecule and shaping post-transcriptional networks [31].

RNA modifications represent another important mechanism of post-transcriptional regulation, influencing mRNA metabolism and shaping immune responses. Among these, N6-methyladenosine (m^6^A) regulates mRNA fate through the reversible deposition and removal of methyl marks, installed by the METTL3–METTL14 methyltransferase complex and erased by demethylases such as FTO and ALKBH5. These modifications are recognized by specific reader proteins, including YTH domain-containing proteins (YTHDF1–3 and YTHDC1), which determine whether an mRNA is efficiently translated, transiently stored, or targeted for degradation. The dynamic regulation of m^6^A in response to immune stimuli allows rapid adjustment of the stability and translation of immune-related mRNAs, enabling immune responses to be swiftly activated and appropriately terminated [24].

Spatial compartmentalization of immune-related mRNAs adds an additional mechanism of post-transcriptional regulation by controlling where and when these transcripts are translated or degraded. During cellular stress, infection, or immune activation, mRNAs encoding cytokines and other inflammatory mediators can be selectively sequestered into cytoplasmic structures such as stress granules (SG), where their translation is temporarily repressed, or processing bodies (P-bodies), where they are directed toward decay pathways. Alternately, immune-related mRNAs are localized to specific subcellular regions that favor efficient translation. Through this spatial organization, cells can precisely coordinate mRNA translation and turnover, and thereby refine both the magnitude and the timing of inflammatory responses and prevent inappropriate or prolonged immune activation [26,32,33].

Among post-transcriptional regulators, the CCCH-type zinc finger protein family, particularly the ZFP36 family, plays a critical role in immune regulation. These proteins selectively bind AU-rich element (ARE)-containing transcripts and recruit components of the mRNA decay machinery, leading to the rapid downregulation of pro-inflammatory mediators [34]. The selective targeting of these RNA-binding proteins may offer an effective therapeutic approach for modulating inflammatory responses to improve pathogen control or to treat inflammatory disorders [35].

## 4. Discovery and Structural Characteristics of the ZFP36 Protein Family Members

The ZFP36 protein family, commonly referred to as the tristetraprolin (TTP) family, is named after its initial structure, which is characterized by tandem CCCH-type zinc-finger motifs, each coordinated by four conserved zinc-binding residues (Cys_3_His), and by flexible proline-rich regions that flank the RNA-binding domain [36,37,38]. The first protein containing a CCCH-type zinc-finger domain was originally identified in *Xenopus laevis* oocytes as transcription factor IIIA (TFIIIA), where it was initially described as a transcriptional regulator involved in 5S rRNA gene expression [39,40]. Subsequent biochemical and structural analyses revealed that the CCCH motif also functions as an RNA-binding module, fundamentally revising the understanding of zinc-finger proteins as regulators extending beyond transcriptional control [41,42].

Mammalian ZFP36 has approximately 326 amino acids with conserved RNA-binding core [42,43]. The RNA-binding core or tandem zinc-finger region in the protein consists of two CCCH motifs, each coordinating a single zinc ion through four conserved residues arranged in a Cys–Cys–Cys–His configuration [37,42,43]. High-resolution solution structural studies demonstrated that these zinc fingers adopt a defined fold and function cooperatively to recognize AU-rich elements within single-stranded RNA, providing direct structural evidence for RNA binding by CCCH zinc fingers [44]. Structural studies revealed conserved aromatic and hydrophobic residues, along with basic amino acids positioned to contact RNA bases and the phosphate backbone. Zinc coordination by conserved cysteine and histidine residues stabilizes the tandem zinc-finger fold required for high-affinity recognition of AU-rich elements (AREs) [37,45].

The ZFP36 family in mammals includes four members such as ZFP36 (TTP), ZFP36L1, ZFP36L2, and ZFP36L3, with ZFP36L3 being present only in rodents [46]. All family members have a highly conserved structural core made up of two tandem CCCH-type zinc finger motifs that are required for RNA binding and ARE recognition [36].

ZFP36, ZFP36L1, and ZFP36L2 share a conserved tandem zinc-finger (TZF) domain, with more than 70% amino-acid sequence identity within the RNA-binding region; however, they differ substantially outside this core. The N- and C-terminal flanking regions show much lower amino-acid sequence identity, which is estimated at approximately 10–24% [47]. When comparing ZFP36L1 and ZFP36L2, ZFP36L1 is a relatively compact protein of approximately 338 amino acids, whereas ZFP36L2 is considerably larger, comprising about 494 amino acids. ZFP36L1 contains shorter N- and C-terminal regions, while ZFP36L2 has extended low-complexity regions enriched in proline, glutamine, alanine, and serine residues, which are commonly post-translationally modified and likely contribute to functional and regulatory differences between ZFP36L1 and ZFP36L2 [47,48,49]. Unlike ZFP36, ZFP36L1, and ZFP36L2, ZFP36L3 is a rodent-specific paralogue that retains the conserved tandem zinc-finger (TZF) RNA-binding domain but exhibits greater divergence within its N- and C-terminal regions. Consistent with its restricted expression in placental and yolk sac tissues, this structural divergence is thought to contribute to its specialized role in extraembryonic development [50].

All three ZFP36 family members contain nuclear localization signals (NLS) and nuclear export signals (NES), enabling them to shuttle between the nucleus and cytoplasm. In ZFP36, nuclear export is mediated by a leucine-rich NES located in the N-terminal region and occurs through a CRM1 (exportin-1)-dependent pathway, while nuclear import is mediated by an NLS located near the tandem zinc-finger (TZF) domain. In contrast, ZFP36L1 and ZFP36L2 contain NES motifs in their C-terminal regions and similarly rely on CRM1-dependent nuclear export. ZFP36L1 is distinguished by a serine-rich C-terminal regulatory region that controls cell-cycle-dependent nuclear accumulation, whereas ZFP36L2 possesses extended low-complexity terminal regions that favor cytoplasmic localization. Despite a highly conserved TZF RNA-binding domain, divergence within the flanking regions is likely to drive paralogue-specific regulatory functions and biological roles [49].

**Figure 1:**
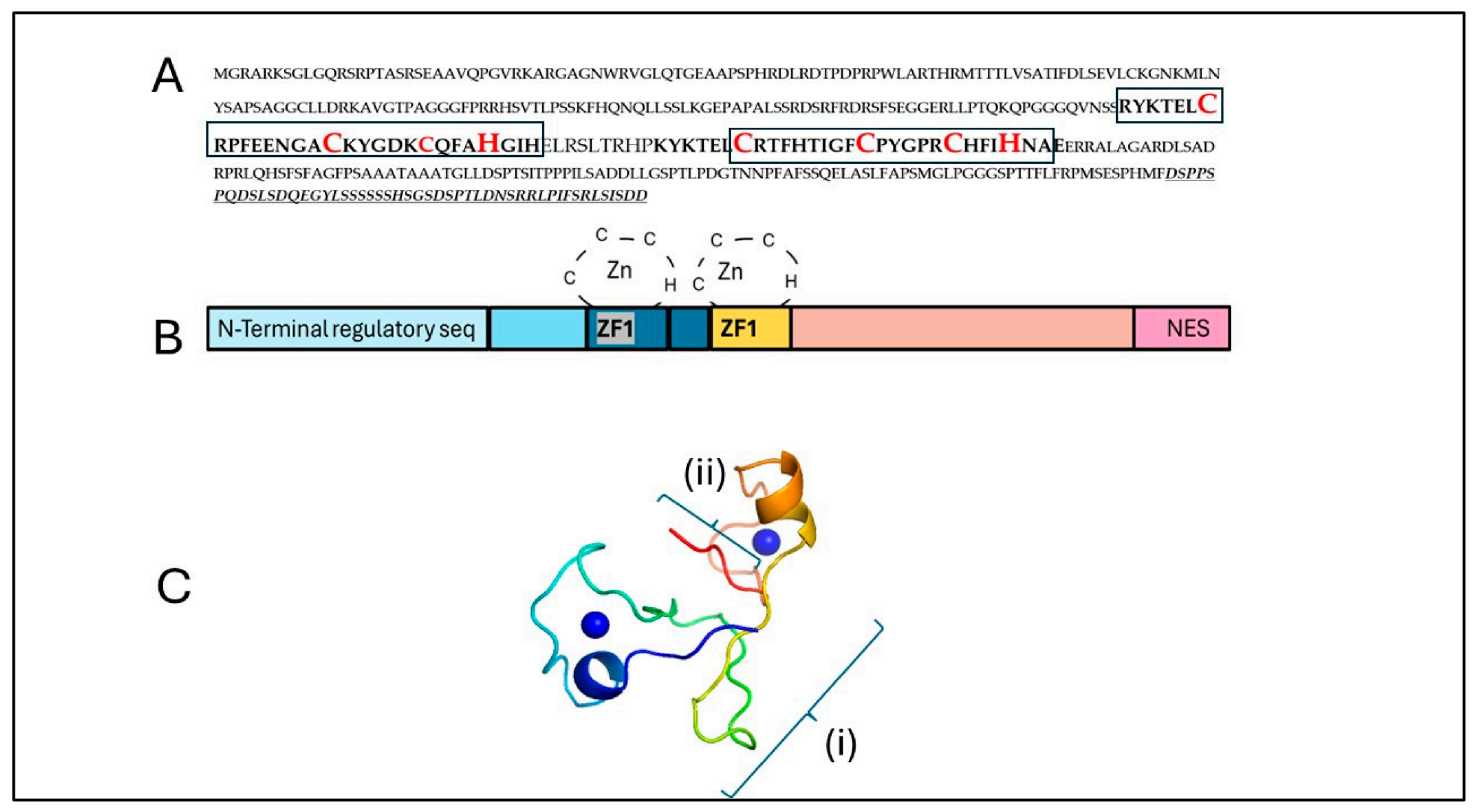
Homology model and domain organization of ZFP36L1. The mRNA sequence of human (*Homo sapiens*) ZFP36L1 was retrieved from the NCBI database (accession number NM_001244701.1). The mRNA sequence was translated into the corresponding protein sequence using the ExPASy Translate tool. A homology model of the ZFP36L1 protein was generated using the SWISS-MODEL server. (**A**) Protein sequence highlighting the conserved tandem zinc finger domains characterized by the motif Cys–X_8_–Cys–X_5_–Cys–X_3_–His. (**B**) Schematic linear representation of the ZFP36L1 protein showing the domain organization. (**C**) Homology model of the ZFP36L1 protein structure generated using SWISS-MODEL. The zinc finger domains coordinating Zn^2+^ ions are highlighted in blue. These domains also contain the nuclear localization signal (NLS) indicated by (**i**), while the C-terminal nuclear export signal (NES) is indicated by (**ii**).

**Figure 2:**
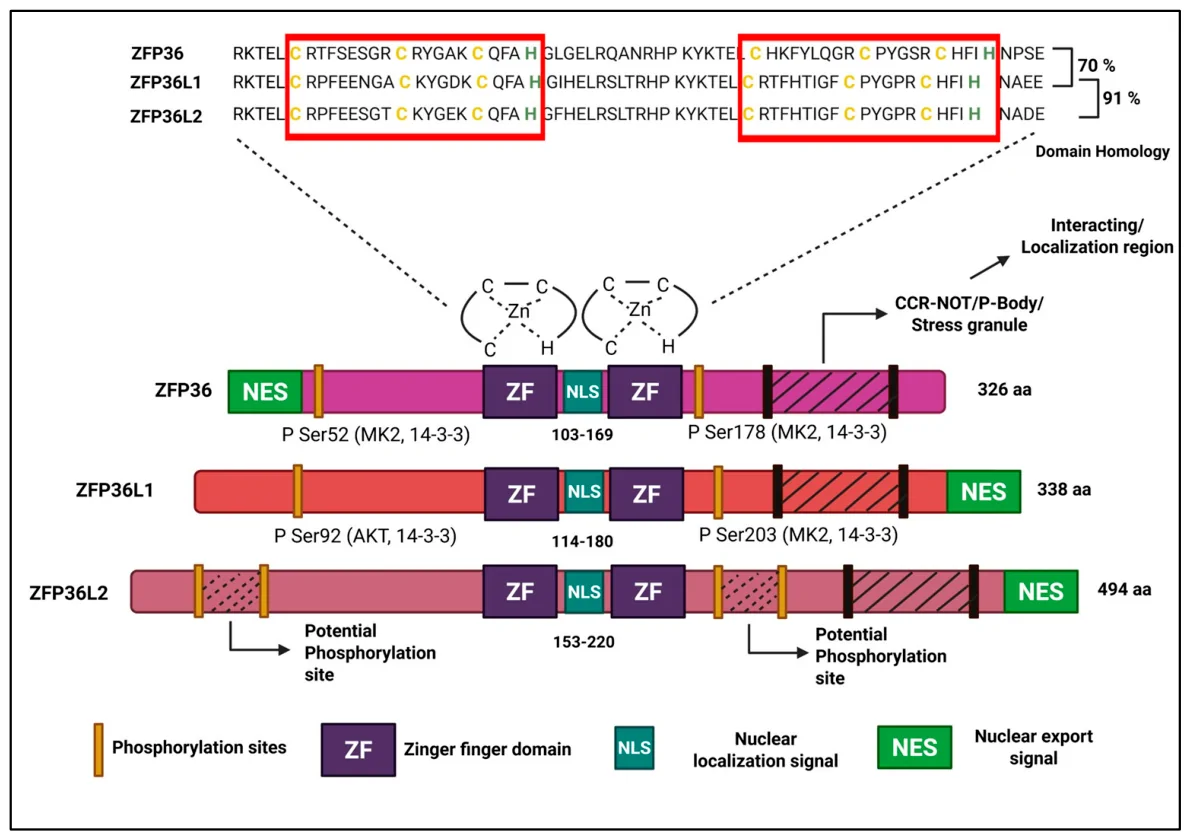
Comparative domain architecture and functional features of ZFP36 family proteins. Schematic comparison of the domain architecture of ZFP36 (tristetraprolin; TTP), ZFP36L1 (formerly butyrate response factor 1; BRF1), and ZFP36L2 (formerly butyrate response factor 2; BRF2). All three proteins contain a highly conserved tandem CCCH zinc-finger (TZF) RNA-binding domain that recognizes AU-rich element (ARE)-containing mRNAs. The amino acid sequence alignment (top) illustrates the high degree of conservation within the TZF domains. Conserved nuclear localization signals (NLS), nuclear export signals (NES), experimentally validated phosphorylation sites (ZFP36 Ser52 and Ser178; ZFP36L1 Ser92 and Ser203), and predicted phosphorylation sites in ZFP36L2. The C-terminal interacting/localization region is associated with interactions with the CCR4–NOT deadenylase complex, processing bodies (P-bodies), and stress granules involved in mRNA decay. Protein lengths are shown on the right, and the percentage sequence homology within the TZF domain. (Figure modified from previously published article [50]).

In addition to differences in nuclear–cytoplasmic trafficking and protein length, members of the ZFP36 family display distinct expression patterns, regulatory mechanisms, and biological functions, which are discussed in greater detail in the following sections. Briefly, ZFP36 is constitutively expressed at low levels in multiple cell types but is rapidly induced by inflammatory and stress stimuli, where it promotes the degradation of cytokine and inflammation-associated mRNAs, including *tumor necrosis factor-alpha TNFα* [34,42]. In contrast, ZFP36L1 regulates transcripts involved in cell-cycle progression, cellular quiescence, genome stability, embryonic development, and antiviral responses, reflecting broader biological functions beyond inflammatory mRNA regulation [51]. ZFP36L2 is predominantly expressed within the hematopoietic compartment, particularly in developing lymphocytes and thymocytes, where it cooperates with ZFP36L1 to regulate lymphocyte differentiation, cellular quiescence, and adaptive immune development [51,52]. The principal similarities and differences among ZFP36 family members with respect to structural organization, expression pattern, nucleocytoplasmic trafficking, immune regulation, and biological function are summarized in Figure 1 and Figure 2, together with Table 1.

Although all three proteins bind AU-rich elements and recruit mRNA decay machinery, they differ in the strength and context of their RNA-destabilizing activity. ZFP36 is generally the most potent inducer of rapid mRNA decay, whereas ZFP36L1 and ZFP36L2 often act in a more regulated, context-dependent manner that is strongly influenced by phosphorylation and subcellular localization [34,47]. These functional differences are further reinforced by divergence in the N- and C-terminal regions, which confer distinct post-translational modification patterns and protein–protein interaction networks, including differential engagement with the CCR4–NOT deadenylase complex [42,52].

## 5. Baseline Expression of ZFP36 Family Protein Members

Baseline expression data show that members of the ZFP36 family share some common expression features but also display clear paralogue-specific patterns that reflect their different biological roles. Large-scale RNA-seq datasets from Genotype-Tissue Expression (GTEx) and the Human Protein Atlas indicate that ZFP36 (TTP) is expressed at low to moderate levels across many human tissues, including the lung, liver, spleen, thymus, and intestine, with particularly strong expression in hematopoietic and immune-related tissues [74]. Single-cell RNA-seq studies further demonstrate that ZFP36 transcripts are consistently present in circulating monocytes and tissue-resident macrophages, where expression levels rank among the higher group of immune-associated RNA-binding proteins [13]. Although absolute expression levels vary between datasets due to technical differences, the overall pattern is consistent and shows a clear bias toward immune cells.

In contrast to ZFP36, the related family members ZFP36L1 and ZFP36L2 show broader, more constant expression beyond classical immune tissues. ZFP36L1 is expressed across a wide range of tissues, including skin, bone marrow, lymphoid organs, and the nervous system. While ZFP36L2 is expressed in lymphoid progenitors, hematopoietic stem and progenitor cells and intestinal crypts [57,58,59,60,61,62,63,64,65,66,67,68,69,70,71,72,73,74,75,76,77,78,79]. This widespread expression pattern is consistent with their roles in regulating the cell cycle, supporting lymphocyte development, and maintaining quiescent progenitor cell states, rather than driving acute inflammatory responses. By contrast, ZFP36L3 is largely restricted to rodents and is highly expressed in placental and extra-embryonic tissues in mice, with no functional counterpart in humans [80]. This suggests that ZFP36L3 has evolved specialized roles in placental mRNA regulation.

## 6. Cytoplasmic-Nuclear Shuttling of ZFP36 Family Proteins

ZFP36 family proteins undergo dynamic shuttling between the nucleus and cytoplasm, a regulatory feature that controls access to target mRNAs and their involvement in mRNA turnover. Studies have shown that ZFP36 family proteins contain both nuclear localization (NLS) sequences located within the tandem zinc finger domain and hydrophobic amino acid-rich nuclear export signals (NES) at N-terminal region, enabling active transport across the nuclear envelope through CRM1-dependent export pathways [46,57]. Under basal conditions, ZFP36 family proteins are distributed between the nucleus and cytoplasm; however, inflammatory or stress stimuli promote their cytoplasmic accumulation, positioning them alongside the cytoplasmic mRNA decay machinery [46,81].

Post-translational modifications, particularly phosphorylation, play a key role in this redistribution or functional activation. MK2-mediated phosphorylation promotes cytoplasmic retention of ZFP36 proteins and increases its interaction with 14-3-3 proteins, adaptor proteins that bind phosphorylated serine or threonine motifs on target proteins, thereby reducing its localization to stress granules and P-bodies, which are major sites of translational repression and mRNA decay [82,83]. This mechanism enables cells to transiently pause ZFP36 engagement with AU-rich element (ARE)-containing transcripts, thereby fine-tuning cytokine mRNA stability during immune activation.

Differences in nucleocytoplasmic distribution further expand the functional range of the ZFP36 family. ZFP36L1 and ZFP36L2 show stronger nuclear enrichment during specific biological processes such as lymphocyte development and cell-cycle regulation, suggesting their roles are not limited to cytoplasmic mRNA decay [81]. Studies have showed that their nuclear role influences gene expression at earlier regulatory stages, potentially by interacting with transcriptional machinery or chromatin-associated complexes. Such activities would allow ZFP36L1 and ZFP36L2 to coordinate transcriptional control with post-transcriptional mRNA regulation, thereby integrating multiple layers of gene expression control [81,84]. ZFP36L1 and ZFP36L2 help maintain cellular quiescence and restrict cell-cycle progression and DNA damage responses at key developmental checkpoints [61,77,78]. Their specific roles in B- and T-cell development are discussed in a separate section.

Together, nucleocytoplasmic trafficking highlights that the ZFP36 family possesses an evolutionarily flexible regulatory feature that integrates with phosphorylation. This spatial control allows ZFP36 family members to rapidly adjust their functional availability by engaging or disengaging from target/transcripts in response to inflammatory and developmental conditions and maintain cellular homeostasis [57,61,75].

## 7. Role of ZFP36 Protein Family in Controlling Pro-Inflammatory Cytokines

Members of the ZFP36 family are rapidly induced in response to cellular stress and immune activation, acting as early regulators of gene expression. Their rapid upregulation allows timely control of ARE- containing transcripts, including those encoding inflammatory mediators, thereby limiting stress and immune-driven gene expression [11,62,85]. Conversely loss of ZFP36 results in excessive production of TNF-driven inflammatory syndromes [62,64].

In macrophages, ZFP36 is rapidly upregulated following Toll-like receptor (TLR) engagement, functioning as a negative feedback regulator of excessive cytokine production such as TNF-α and IL-6, thereby preventing hyperinflammatory responses [34,63]. ZFP36 acts as a key component of a negative feedback loop that regulates TNF-α production by destabilizing *TNF-α* mRNA. Specifically, ZFP36 downregulates TNF-α expression by directly binding to its AREs within the 3′ UTR of *TNF α* mRNA and promoting its decay through recruitment of the CCR4–NOT deadenylase complex [86]. ZFP36 also use similar ARE-dependent mechanism to suppression of IL-6 expression [87].

Beyond TNF-α and IL-6, ZFP36 and its family members (ZFP36L1 and ZFP36L2) regulate a broad spectrum proinflammatory/inflammatory cytokines, including IL-12, IL-23, IL-2, IL-8, GM-CSF/CSF2, cyclooxygenase-2 (COX2) and IFN-γ [42,88,89,90,91,92]. This regulation occurs either through AU-rich element (ARE)-dependent post-transcriptional mRNA decay or indirectly by modulation of cytokine expression by suppressing their transcription factor NF-κB.

ZFP36 family proteins control cytokines through a negative feedback mechanism that has been well summarized in previous reviews [13]. Briefly, members of the ZFP36 family, including ZFP36L1, tightly regulate cytokine expression by coordinating NF-κB–dependent transcriptional activation and phosphorylation-regulated post-transcriptional mRNA decay mechanisms [13]. Upon Toll-like receptor (TLR) stimulation by specific ligands, such as lipopolysaccharide (LPS) for TLR4, activation of the IκB kinase (IKK) complex leads to phosphorylation and degradation of IκBα, thereby facilitating NF-κB translocation to the nucleus and induction of proinflammatory cytokine genes, including *TNFα* [93,94]. Concurrently, ZFP36 family proteins restrict excessive cytokine production at the post-transcriptional level by binding AREs within the 3′ untranslated regions (UTRs) of target mRNAs encoding cytokines and promoting their decay through recruitment of the CCR4–CAF1–CNOT1 deadenylase complex. The detailed molecular mechanisms underlying this process are discussed in a subsequent section. The efficacy of ZFP36 family proteins in controlling cytokine expression is tightly regulated by two major well-established mechanisms

(i) Phosphorylation-dependent regulation: Activation of the p38 MAPK pathway leads to MK2-mediated phosphorylation of ZFP36 family members at conserved serine residues, promoting their association with 14-3-3 proteins and thereby inhibiting ARE-mediated mRNA decay, resulting in transient stabilization of cytokine transcripts [57]. Termination of this response requires dephosphorylation of ZFP36 family proteins by serine/threonine phosphatases such as PP2A, while the dual-specificity phosphatase DUSP1 attenuates upstream p38 MAPK signaling. Through this reversible phosphorylation-dependent switch, ZFP36 family proteins precisely regulate NF-κB–driven inflammatory responses [13,82,95].
  Additionally, ERK–RSK–mediated phosphorylation near the C-terminus of ZFP36L1 and ZFP36L2 has been shown in non-immune cells to reduce their interaction with the CCR4–NOT complex, thereby diminishing their mRNA-destabilizing activity [96].
(ii) Autoregulatory control: ZFP36 also autoregulates its own expression by binding to AREs within the 3′ UTR of its mRNA, thereby establishing an additional negative feedback loop that maintains inflammatory signaling [13,97].

Phosphorylation-dependent regulation and autoregulatory control of the ZFP36 family are tightly integrated with additional mechanisms, including regulation of ZFP36 protein stability, subcellular localization, competition with stabilizing RNA-binding proteins, microRNA-mediated repression/cooperation, and cell-type–specific transcription. For example, dephosphorylated ZFP36 is more susceptible to ubiquitin-mediated proteasomal degradation, whereas phosphorylation by the p38 MAPK–MK2 pathway stabilizes ZFP36 protein. This coordinated regulation allows ZFP36 proteins’ stability and activity and regulates inflammatory response [98,99]. ZFP36 family proteins also are localized to stress granules (SG) or cytoplasmic processing bodies (P-bodies), which are the sites of mRNA storage and decay. MK2-mediated phosphorylation prevents ZFP36 association with these compartments, thereby reducing access to target mRNAs and attenuating mRNA decay [82].

Cell-type–specific transcriptional regulation also governs the activity of the ZFP36 protein family. The expression levels and functional dominance of ZFP36 family members vary across cell types, with ZFP36 predominating in myeloid cells, whereas ZFP36L1 and ZFP36L2 play more prominent roles in lymphocytes as mentioned above. This differential transcriptional regulation enables lineage-specific control of cytokine expression and immune responses [55,58,74,78,80,100,101].

ZFP36 is subject to microRNA-mediated regulation. MicroRNA, miR-29a has been shown to directly target the 3′ UTR of ZFP36 mRNA, leading to reduced ZFP36 expression [102]. In contrast, miR-16 does not directly target ZFP36 but functionally cooperates with it in ARE-mediated mRNA decay, thereby contributing the cytokines transcripts [103]. Furthermore, the ability of ZFP36 family proteins to bind AREs within immune mediator transcripts can be functionally antagonized by stabilizing RNA-binding proteins, such as HuR (ELAVL1), which compete for ARE binding and promote mRNA stabilization [104]. Anti-inflammatory cytokines also utilize members of the ZFP36 family to suppress inflammatory responses. For example, IL-10 induces ZFP36 expression through a STAT3-dependent pathway. Increased ZFP36 expression promotes the decay of proinflammatory cytokine mRNAs, thereby limiting downstream inflammatory gene expression [105].

In addition to promoting mRNA decay, ZFP36 protein also helps suppress translation. ZFP36 proteins can repress translation independently of mRNA degradation. For example, ZFP36 proteins recruits the 4EHP–GIGYF2 cap-binding complex through conserved proline-rich motifs, thereby inhibiting the translation of target mRNAs [106,107]. Collectively, these mechanisms enable precise temporal and spatial regulation of inflammatory mRNA decay and ensure balanced cytokine responses during immune activation [102,103,104,106,107,108].

As mentioned above, the defining feature of ZFP36 protein family is the presence of two tandem CCCH-type zinc finger motifs that enable selective, high-affinity RNA binding. Each motif follows the conserved sequence Cys-X_8_-Cys-X_5_-Cys-X_3_-His, where cysteine and histidine residues coordinate a zinc ion to form a stable structure. This structure allows ZFP36 proteins to recognize AREs within the 3′ UTRs of target mRNAs [109]. Previous studies with zinc finger proteins showed that zinc ions are crucial for their functional activity and substituting zinc with other metals such as iron (Fe), copper (Cu), cadmium (Cd), or gold (Au) significantly impairs zinc finger proteins’ RNA-binding capacity [110,111].

The tandem zinc finger domains of ZFP36 proteins enable cooperative, high-affinity binding to UUAUUUAUU motifs [112], a conserved consensus sequence frequently found in cytokine and immune-regulatory mRNAs. Upon binding, ZFP36 recruits components of the mRNA decay machinery, including the CCR4–NOT deadenylase complex and decapping enzymes, thereby promoting rapid degradation of target transcripts [113]. Structural analysis has shown that even single amino acid substitutions within the zinc finger motifs can disrupt RNA binding capacity, leading to impaired mRNA decay, dysregulated cytokine expression, and subsequent inflammatory pathology [114].

Structural studies have shown that ZFP36 family proteins contains a conserved C-terminal motif that directly interacts with CNOT1, the scaffold subunit of CCR4–NOT, thereby facilitating deadenylase recruitment to the bound mRNA and promoting poly(A) tail removal [115]. Deadenylation makes the mRNA susceptible that undergo further degradation. The transcript with short poly A tail first be decapped by the DCP1–DCP2 complex, which removes the protective 5′ cap structure. Once decapped, the mRNA is degraded in the 5′–3′ direction by the exonuclease XRN1. Alternatively, degradation can proceed from the 3′ end through the RNA exosome complex [17,116,117,118].

## 8. Role of the ZFP36 Family in Immune Cell Differentiation, Development, and Migration

The ZFP36 family of RNA-binding proteins, including ZFP36 (TTP), ZFP36L1, and ZFP36L2, play critical roles in innate and adaptive immune cells differentiation and development [16,56,61,90] as well as their trafficking [119,120,121,122,123]. Studies have demonstrated that ZFP36 regulates the expression of chemokines and chemokine receptors, including CXCL1 [119,120,121], CXCL2 [120], CCL3 [124] and CXCR4 [122], thereby modulating neutrophil and macrophage trafficking and contributing to maintaining tissue homeostasis.

The ZFP36 protein has also been shown to regulate the lifespan of activated neutrophils during infection. Under homeostatic conditions, ZFP36 does not affect overall neutrophil numbers; however, it promotes apoptosis in activated, tissue-infiltrating neutrophils, thereby limiting excessive inflammation. In contrast, ZFP36-deficient neutrophils remain activated for a prolonged duration. Mechanistically, the reduced apoptosis observed in ZFP36-deficient neutrophils was associated with increased stabilization of *Mcl1* which encodes myeloid cell leukemia-1, an anti-apoptotic protein [123].

The ZFP36 family plays an important role in early thymocyte development as well as in controlling the activity of mature immune cells. Early thymocyte development is governed by stringent developmental checkpoints. At the DN3 (double-negative 3) stage, productive rearrangement of the TCRβ chain triggers β-selection, initiating proliferation and differentiation toward the DN3b and subsequent Double-positive thymocyte (DP) stages. This proliferation must be tightly controlled to prevent expansion of cells with incomplete or aberrant TCR rearrangements.

ZFP36L1 and ZFP36L2 play a critical regulatory role at this checkpoint. Mice with T cell-specific deletion of both *Zfp36l1* and *Zfp36l2* (via CD2-Cre) bypass β-selection, allowing DN3 thymocytes to proliferate in the absence of a functional TCRβ chain. These aberrantly expanding cells progress toward T cell acute lymphoblastic leukemia (T-ALL). In contrast to T cells, deletion of both *Zfp36l1* and *Zfp36l2* in B cells did not result in B-cell malignancies but instead led to increased B-cell apoptosis [56].

During early B cell development, pro-B cells sequentially progress to pre-B cells as they mature. At these stages, cells undergo tightly regulated transitions between proliferation and V(D)J recombination. Periods of quiescence are essential in both of these stages before progression because V(D)J recombination involves RAG-mediated DNA double-strand breaks that must be repaired before next stage of cell development. Cells must remain in G0/G1 phase during this process. Premature progression from G1 into S phase during active recombination can propagate unrepaired DNA which impair immunoglobulin gene assembly and trigger apoptosis [125,126,127]. ZFP36L1 and ZFP36L2 are essential for maintaining this quiescent state during critical recombination windows. In B cell specific double knockout mice (negative for both ZFP36L1 and ZFP36L2), developing B cells entered the cell cycle prematurely, leading to defective immunoglobulin gene recombination, and increased apoptosis [61,128].

The spleen is organized into distinct regions, including the red pulp and the white pulp. The white pulp contains B cell follicles, which are divided into follicular (FO) B cells and marginal zone (MZ) B cells [129]. FO B cells recirculate and participate in germinal center responses, whereas MZ B cells reside at the outer edge of the white pulp and provide rapid antibody responses to blood-borne pathogens and antigens [130]. ZFP36L1 has a distinct role in maintaining the MZ B cell population. When ZFP36L1 was conditionally deleted in B cells (while ZFP36L2 remained intact), the MZ B cell subset collapsed [15,131]. Transcriptomic analysis revealed increased expression of *Klf2* and *Irf8*, two encodes transcription factors that regulate B cell positioning and fate [15]. KLF2 controls cell trafficking by inducing expression of migration and homing receptors such as S1PR1 and CD62L (L-selectin). These molecules promote B cell circulation to secondary lymphoid organs and facilitate lymphocyte egress from spleen [131]. In contrast, IRF8 regulates B cell lineage decisions by controlling transcriptional programs that influence differentiation and activation [132]. Together, these findings indicate that ZFP36L1 restrains expression of key transcription factors such as KLF2 and IRF8 to preserve proper positioning and identity of marginal zone B cells [15].

In addition to immune cell development, ZFP36 proteins also play critical roles in modulating mature immune cells. In mature T cells, ZFP36 limits T-cell activation and effector differentiation by binding to and promoting the degradation of mRNAs encoding key immune mediators such as NF-κB1, c-Rel, IL-2, IFN-γ, and TNF. While loss of ZFP36 results in accelerated T-cell activation and increased proliferation [71].

Another study showed that ZFP36 and ZFP36L1 together regulate the timing of CD8^+^ T cell differentiation. These proteins slow the differentiation of activated naïve CD8^+^ T cells and limit the potency of differentiated cytotoxic lymphocytes by suppressing mRNAs that encode key transcription factors, including NF-κB, IRF8, and Notch1, as well as cytokines such as IL-2, which are important for T cell activation. When both ZFP36 and ZFP36L1 are deleted in T cells, CD8^+^ T cells activate and differentiate more rapidly and develop into more potent cytotoxic cells. Mice receiving small numbers of these double-deficient CD8^+^ T cells show improved protection against influenza A virus infection without increased immunopathology [133].

ZFP36L1 also regulates the migration and localization of plasma cells (antibody-secreting cells, ASCs). Studies have shown that in mice lacking ZFP36L1 in B cells, early plasmablast responses are normal; however, the homing of ASCs to the bone marrow is impaired. As a result, ASCs accumulate in the spleen and liver instead of migrating efficiently to the bone marrow, where long-lived plasma cells normally reside [134].

## 9. ZFP36 Family–Autophagy Relationship

In addition to regulating immune cell differentiation and inflammatory responses, ZFP36 family proteins are also involved in the regulation of autophagy, a process that plays an important role in immune defense and antiviral responses. Autophagy is a conserved lysosomal degradation process that removes damaged organelles, recycles cellular components, and contributes to immune defense by eliminating intracellular pathogens [135,136,137]. Autophagy also regulates antigen presentation and inflammasome activation, highlighting its importance in both innate and adaptive immunity [137,138,139]. Several studies suggest that ZFP36 family proteins play important roles in the regulation of autophagy. Evidence indicates that ZFP36 can either promote or inhibit autophagy, acting directly through modulation of autophagy-related gene expression or mRNA stability, or indirectly by affecting immune signaling pathways, cellular metabolism, and stress-response mechanisms. Nevertheless, additional studies are needed to better understand these mechanisms and establish definitive conclusions.

A study reported that AUF1 and ZFP36L1 promote the destabilization of *Bcl-2* mRNA in macrophages, resulting in reduced Bcl-2 levels and increased Beclin-1 activity, which facilitates the activation of autophagy [66].

In contrast, another study showed that ZFP36 inhibits ferroptosis in hepatic stellate cells (HSCs) by destabilizing *ATG16L1* mRNA and reducing autophagy [68]. Similarly, a study in lung adenocarcinoma cells demonstrated that ZFP36 expression is significantly reduced in these cells. Overexpression of ZFP36 resulted in a decrease in autophagy, as indicated by reduced levels of the autophagy markers Beclin1 and the LC3-II/LC3-I ratio. This reduction in autophagy markers was associated with ZFP36-mediated suppression of the NF-κB signaling pathway. These findings suggest that ZFP36 regulates autophagy largely through modulation of NF-κB signaling in lung adenocarcinoma cells [140].

There are indirect relationships indicating that ZFP36 may suppress autophagy through stress-related RNA regulation mechanisms. Stress granules (SGs) often interact with P-bodies (processing bodies), which are cytoplasmic structures that contain enzymes involved in mRNA degradation and turnover. P-bodies are enriched with decay factors such as DCP1/2, XRN1, and the CCR4-NOT complex, which remove the mRNA cap, degrade transcripts, or shorten poly(A) tails. Through interactions between SGs and P-bodies, mRNAs can move between temporary storage in stress granules and degradation in P-bodies, allowing cells to regulate gene expression during stress [141,142,143]. If stress granules persist for too long or fail to disassemble properly, cells activate granulophagy, a selective form of autophagy that removes damaged or long-lived RNA–protein aggregates. In this process, ubiquitinated stress-granule proteins such as G3BP1 (Ras GTPase-activating protein-binding protein 1), a key stress granule nucleating protein that promotes SG assembly, are recognized by autophagy receptors including p62/SQSTM1 and NDP52, which connect these aggregates to LC3-positive autophagosomes for lysosomal degradation. In this way, autophagy acts as an important quality-control system that clears abnormal RNA protein complexes [144,145]. Within this RNA regulatory network, ZFP36 family proteins serve as important regulators of mRNA turnover. These proteins localize to both stress granules and P-bodies, where they promote mRNA turnover. By facilitating efficient RNA degradation and preventing persistent stress granules, ZFP36 proteins may indirectly limit excessive activation of autophagy [146].

Collectively, these findings indicate that ZFP36 family proteins play important roles in regulating autophagy through direct modulation of autophagy-related genes as well as indirect regulation of cellular stress response pathways. Given the complex role of autophagy during viral infection, where it serves as an important innate defense mechanism but is also frequently exploited by viruses to facilitate their replication, assembly, persistence, or immune evasion, regulation of this pathway by ZFP36 family proteins may represent an additional host-directed antiviral mechanism. The antiviral mechanisms of ZFP36L1, including its autophagy-dependent antiviral activity, are discussed in the following section.

## 10. Role of ZFP36 Family Proteins in Antiviral Defense

All viruses, regardless of whether they possess DNA or RNA genomes, must generate messenger RNA (mRNA) during their replication cycle. Viral mRNAs often structurally and functionally resemble host cellular mRNAs, enabling their efficient recognition by the host translational machinery and allowing proper processing, stability, and translation within infected cells. Because viral mRNA synthesis is central to gene expression and replication, viruses are classified under the Baltimore system according to their genome type and mRNA synthesis pathway [147]. Therefore, RBPs that interact with or regulate host RNA transcription or processing can significantly influence viral RNA replication [148,149].

ZFP36 proteins can bind viral RNAs and promote their degradation or reduce their translation efficiency, thereby restricting viral replication. Indirectly, these proteins regulate the expression of host immune mediators, including cytokines and chemokines, by controlling the stability of their mRNAs, which influences immune cell activation, differentiation, and trafficking and ultimately shapes the overall antiviral immune response. ZFP36L1 has demonstrated antiviral efficacy against various RNA viruses from different families, such as the human coronavirus strain OC43 (HCoV-OC43) [72], flaviviruses including Japanese encephalitis virus (JEV) and dengue virus (DENV) [17], Influenza A virus (IAV) [19], foot-and-mouth disease virus (FMDV) [73], Crimean-Congo hemorrhagic fever virus (CCHFV) [18] and murine norovirus [67].

The antiviral mechanisms of ZFP36L1 vary depending on the specific virus. For instance, it facilitates the decay of viral RNA by degrading the 3′ UTR followed by an exosomal decay pathway, as observed with flaviviruses [17]. In the case of IAV, it suppressed viral proteins and their trafficking [19]. It inhibited viral replication degrading viral proteins such as viral protein 3 (VP3) and VP4 in FMDV [73] and reduction of viral protein activity, such as nucleoprotein function, through direct interaction between the viral protein and ZFP36L1 as in CCHFV [18]. In addition to these direct antiviral activities, ZFP36L1 can indirectly suppress viral replication by modulating host cellular pathways, including autophagy, thereby creating a cellular environment that is less permissive for viral replication [67].

Similar to ZFP36L1, ZFP36L2 has also been reported to exhibit antiviral activity against flaviviruses, including JEV and DENV. Overexpression of ZFP36L2 significantly reduced JEV and DENV replication, whereas shRNA-mediated knockdown of ZFP36L2 increased viral RNA and viral titers. Mechanistically, ZFP36L2 suppresses viral replication by promoting deadenylation of viral RNA poly(A) tails, thereby facilitating RNA decay [150]. However, unlike ZFP36L1, ZFP36L2 predominantly utilizes the XRN1-mediated 5′–3′ RNA decay pathway to promote RNA degradation. In contrast, ZFP36L1 recruits multiple RNA decay machineries, including both XRN1-dependent 5′–3′ decay and the exosome complex responsible for 3′–5′ RNA degradation [17,150].

ZFP36L2 also demonstrates antiviral activity against CCHFV, similar to ZFP36L1. Both proteins function independently as restriction factors, and expression of either ZFP36L1 or ZFP36L2 alone is sufficient to inhibit viral RNA replication [18]. Interestingly, this antiviral activity does not depend on their canonical RNA-binding function that normally targets AU-rich elements to promote mRNA decay. Instead, these proteins (ZFP36L1/ZFP36L2) directly interact with the viral nucleoprotein (N) through protein–protein interactions, thereby impairing viral replication [18]. More recently, ZFP36L2 was identified as an interferon-β-inducible restriction factor against primate lentiviruses that binds the viral Rev protein, blocks the nuclear export of Rev-dependent viral transcripts, and thereby suppresses HIV-1 replication [70].

In contrast to the antiviral roles of ZFP36L1 and ZFP36L2, another member of the family, ZFP36 has been reported to exhibit proviral activity by promoting replication of Senecavirus A (SVA), a member of the *Picornaviridae* family [69]. That study showed that ZFP36 binds to and stabilizes the mRNA encoding the viral capsid protein, thereby enhancing the production of structural proteins required for viral assembly. This function differs from its classical role in promoting the degradation of AU-rich element-containing transcripts. In addition, ZFP36 was found to promote degradation of mitochondrial antiviral-signaling protein (MAVS), a key adaptor in the RIGI-like receptor signaling pathway. Loss of MAVS signaling suppresses the induction of type I interferons, including Interferon beta (IFN-β), thereby weakening the host antiviral response and facilitating viral replication [69].

Beyond the direct interaction of ZFP36L1 with viral RNA or viral proteins, we hypothesize that ZFP36L1 may also restrict viral infection through indirect host-regulatory mechanisms. One potential mechanism involves the regulation of autophagy, autophagy is frequently exploited by many viruses for their efficient replication, assembly, or release [151]. By limiting autophagy-related pathways, ZFP36L1 could reduce the intracellular environment required for efficient viral replication.

Another potential antiviral mechanism of ZFP36L1 involves the regulation of host receptor expression required for viral entry. ZFP36 family proteins regulate gene expression by promoting degradation of ARE-containing mRNAs. Notably, ZFP36L1 and ZFP36L2 have been shown to bind the 3′-UTR of Low-density lipoprotein receptor (LDLR) mRNA and recruit the CCR4–NOT deadenylase complex, resulting in destabilization and degradation of LDLR transcripts [96].

Because LDLR functions as an entry factor for several viruses (Table 2) [152], including Hepatitis C virus (HCV) [153] and Vesicular stomatitis virus [154], reduced LDLR expression could limit viral attachment and entry into host cells. Therefore, ZFP36L1-mediated destabilization of LDLR mRNA may represent an additional host defense mechanism that restricts viral infection.

**Figure 3:**
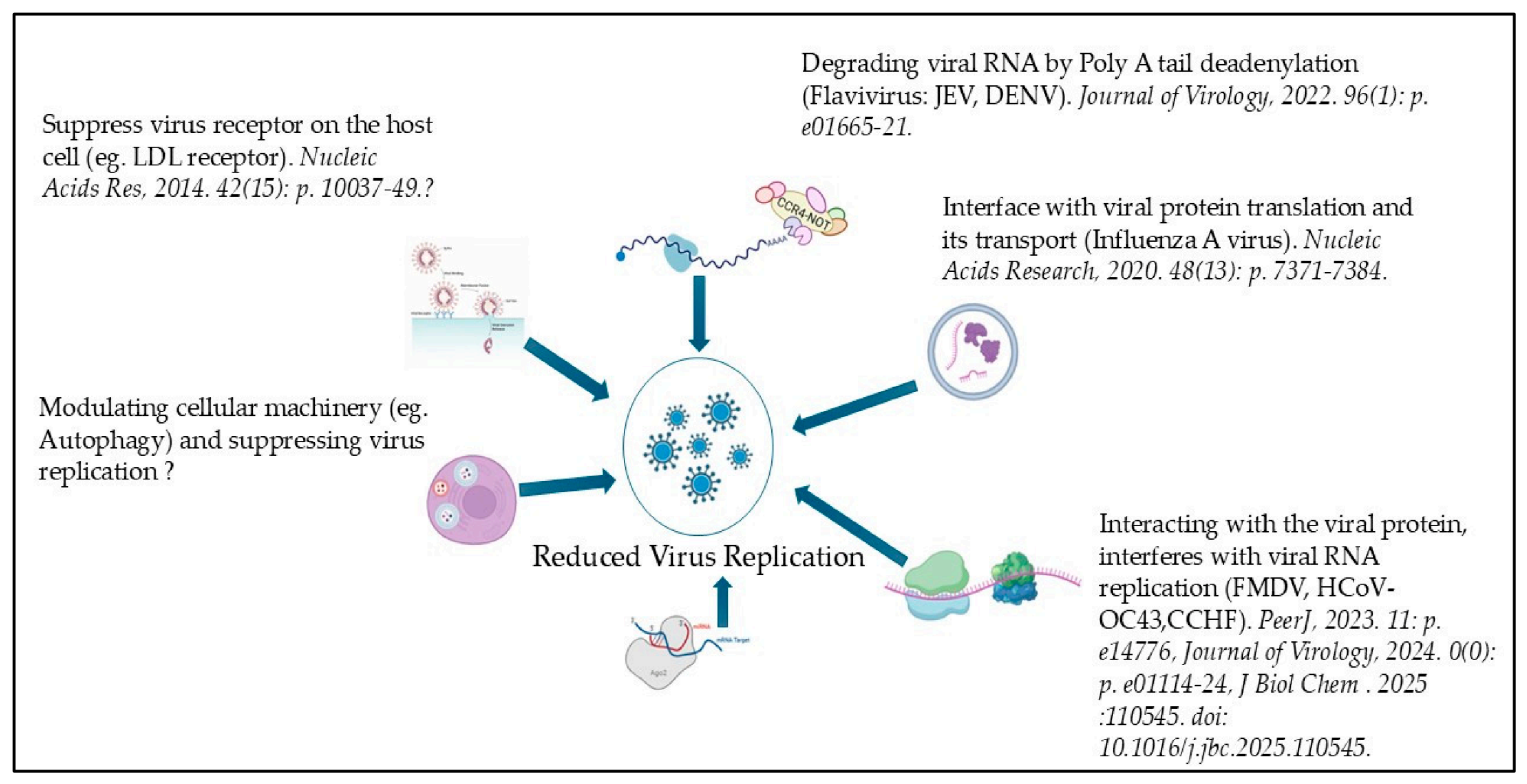
Schematic representation of the broad-spectrum antiviral mechanisms of ZFP36L1. ZFP36L1 suppresses viral replication through distinct mechanisms that vary among virus families. These include viral RNA deadenylation and degradation in flaviviruses (JEV and DENV), inhibition of viral protein translation and trafficking in influenza A virus, direct interaction with viral proteins or transcripts to impair replication in FMDV, HCoV-OC43, and CCHFV, modulation of host cellular pathways such as autophagy, and potentially downregulation of host cell receptors required for viral entry. Together, these complementary activities establish ZFP36L1 as a broad-spectrum antiviral restriction factor that limits viral replication through multiple host-directed mechanisms [17,18,19,72,73,96].

Taken together, these observations suggest that ZFP36L1 may exert broad antiviral effects not only through direct targeting of viral components but also by modulating host cellular pathways, including autophagy and the expression of viral entry receptors, as illustrated in Figure 3.

In addition to its potential role in restricting viral replication, ZFP36L1 may also influence the host inflammatory response, which is a major determinant of disease severity in many viral infections. In several viral diseases, such as infection with SARS-CoV-2 [155] or highly pathogenic influenza viruses [156], host tissue damage is not caused solely by viral replication or the activity of viral proteins but also by dysregulated host immune responses. Severe cases of these infections are frequently associated with hyperinflammation or cytokine storm, which lead to extensive tissue damage and poor clinical outcomes [155,156,157]. Members of the ZFP36 protein family are well-established post-transcriptional regulators of inflammatory gene expression followed by their own expression through feedback mechanisms [97], thereby maintaining inflammatory homeostasis and protecting tissue damage [158].

Because of its dual role in viral inhibition and immune modulation, targeting ZFP36L1 activity could provide both antiviral and immunomodulatory benefits by restricting viral replication while limiting excessive inflammatory responses; however, this concept requires careful evaluation.

## 11. Proposed Mechanistic Model of Host–ZFP36L1/L2–Virus Interactions in Viral Replication

**Figure 4:**
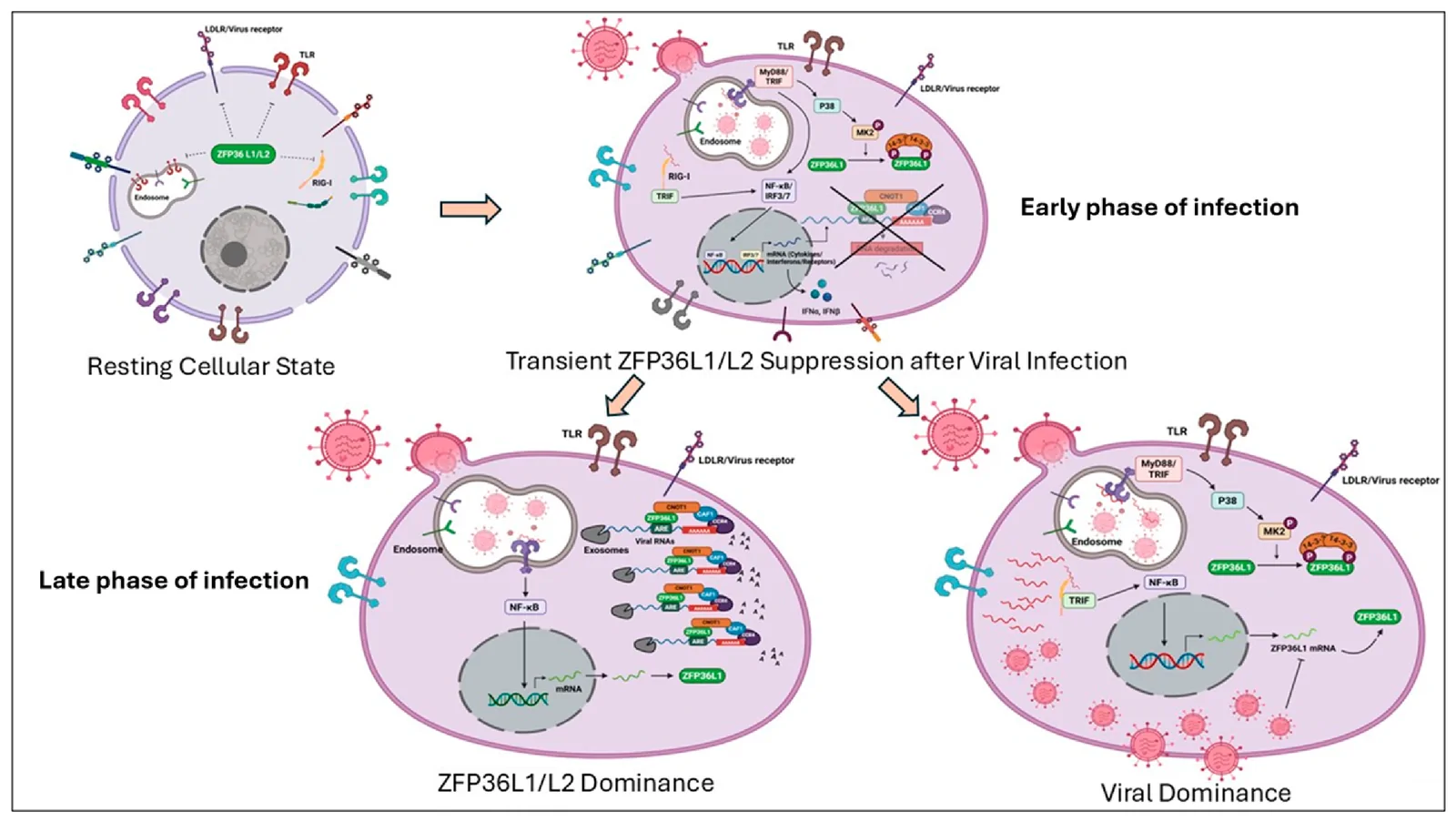
Schematic representation of the proposed mechanistic model of host–ZFP36L1/L2–virus interactions during viral replication. Following viral infection, host pattern-recognition receptors (PRRs) activate innate immune signaling pathways, leading to the induction of type I interferons (IFNs), interferon-stimulated genes (ISGs), inflammatory cytokines, and ZFP36L1/ZFP36L2 expression. During the early phase of infection, activation of the p38–MK2 signaling pathway promotes transient phosphorylation of ZFP36L1/ZFP36L2, reducing their RNA-binding activity through 14-3-3 protein binding. This temporary inhibition is proposed to prevent premature degradation of host antiviral and inflammatory mRNAs, thereby permitting robust expression of interferons and other antiviral effector molecules required for the initial innate immune response. Late stage: ZFP36L1/ZFP36L2 dominance, as infection progresses, dephosphorylation restores the RNA-binding activity of ZFP36L1/ZFP36L2, enabling them to bind AU-rich viral RNA transcripts and/or interact with viral proteins, thereby suppressing viral replication through RNA decay pathways and modulation of host antiviral responses. This later stage can be replaced by viral dominance, some viruses may evade this host defense by suppressing ZFP36L1/ZFP36L2 expression or function.

Based on the evidence described above, ZFP36L1/L2 appears to restrict certain viral infections; however, its role across a broader range of viruses remains largely unexplored. Additionally, the sequential molecular steps involve in host–ZFP36L1/L2–virus interactions, and outcome of these interactions remain largely unknown. We hypothesize the following sequence of events (Figure 4).

Under basal, unstimulated conditions, ZFP36L1/L2 maintain immune homeostasis by regulating the stability of transcripts encoding PRRs and inflammatory mediators. Many immune-related transcripts contain AU-rich elements in their 3′ untranslated regions, making them potential targets of ZFP36 family proteins that promote mRNA decay [159]. Through this mechanism, ZFP36L1/L2 may help maintain low basal levels of PRRs and inflammatory cytokines in the absence of infection, thereby preventing unnecessary immune activation.

During viral infection, viral pathogen-associated molecular patterns (PAMPs) are recognized by host pattern-recognition receptors such as Toll-like receptors (TLRs) and RIG-I–like receptors (RLRs), which initiate innate immune signaling pathways [160]. Viral infection also frequently activates stress-responsive kinase pathways, including the p38 mitogen-activated protein kinase (p38 MAPK) pathway and its downstream kinase MAPK-activated protein kinase 2 (MK2) [161].

Activation of the p38 MAPK/MK2 signaling axis can regulate the activity of ZFP36L1/L2 through phosphorylation, which reduces their ability to destabilize target mRNAs [160,161]. Temporarily suppressing its post-transcriptional mRNA decay activity stabilizes transcripts encoding pro-inflammatory cytokines and antiviral mediators, including type I interferons and pattern recognition receptors (PRRs). As a result, these transcripts accumulate, thereby amplifying the antiviral immune response.

Following this initial phase, the outcome of infection may depend on the nature of the host–ZFP36L1–virus interaction. In some cases, ZFP36L1 activity may remain suppressed due to sustained phosphorylation or viral interference with host regulatory pathways, potentially favoring viral replication or persistence. Alternatively, ZFP36L1 may be dephosphorylated and reactivated, restoring its regulatory activity and limiting viral replication by targeting viral RNAs or viral proteins (Figure 4), However, these proposed mechanisms require experimental validation.

## 12. Conclusions

Post-transcriptional regulation has emerged as a fundamental mechanism for controlling immune responses, enabling rapid and reversible regulation of inflammatory gene expression. Among the key regulatory factors involved in this process, members of the ZFP36 family of RNA-binding proteins play a central role in controlling the stability and translation of AU-rich element (ARE)-containing transcripts. Through this mechanism, they regulate the expression of numerous cytokines and immune-related genes, thereby maintaining immune homeostasis and preventing excessive inflammation. Because cytokine and interferon responses are also critical for shaping vaccine-induced immunity, these regulatory pathways may influence the magnitude and quality of immune responses following vaccination. Understanding the molecular mechanisms regulated by ZFP36L1 is crucial for determining how viral infections influence its expression and how these changes may affect vaccine-induced immunity.

Increasing evidence indicates that the family members ZFP36L1 and ZFP36L2 exhibit broad-spectrum antiviral activity against several RNA viruses from different viral families through distinct underlying mechanisms. These mechanisms include direct interactions with viral RNA or viral proteins, as well as possible indirect modulation of host cellular pathways that influence viral entry or replication.

However, current studies have examined only a limited number of viruses. Further investigations are therefore needed to evaluate the antiviral activity of these proteins against a wider range of viruses, particularly those of major importance to human and animal health, including viruses that cause significant economic losses in livestock and animal production systems, circulate in animal reservoirs, and may contribute to future spillover events.

Future research should also explore the therapeutic potential of targeting ZFP36 family proteins, including the safety and efficacy of strategies aimed at modulating their activity. A deeper understanding of these regulatory pathways may provide new opportunities for the development of antiviral and immunomodulatory therapies.

## Figures and Tables

**Table 1:** Comparison of the structural, molecular, and functional characteristics of ZFP36 family proteins.

Feature	ZFP36 (TTP)	ZFP36L1 (BRF1)	ZFP36L2 (BRF2)	ZFP36L3	References
Species distribution	Human and rodents	Human and rodents	Human and rodents	Rodent-specific	[42,51]
Conserved RNA-binding domain	Tandem CCCH zinc-finger (TZF)	Tandem CCCH zinc-finger (TZF)	Tandem CCCH zinc-finger (TZF)	Tandem CCCH zinc-finger (TZF)	[42]
Primary expression	Rapidly induced in activated macrophages, monocytes, activated T cells, and other leukocytes during inflammation.	Widely expressed in embryonic and adult tissues, including epithelial, endothelial, mesenchymal, and immune cells, with particularly high expression during embryogenesis, placental development, and vascular morphogenesis	Predominantly expressed in the hematopoietic compartment, including hematopoietic stem/progenitor cells, thymocytes, developing B- and T-lymphocytes, and mature lymphoid organs	Restricted to placenta and yolk sac in rodents	[51,53,54,55,56]
Nuclear–cytoplasmic shuttling	CRM1-dependent nucleocytoplasmic shuttling; predominantly cytoplasmic following nuclear export	CRM1-dependent nucleocytoplasmic shuttling; localization regulated by phosphorylation and 14-3-3 binding	CRM1-dependent nucleocytoplasmic shuttling; dynamic localization during lymphocyte development	Predominantly cytoplasmic; nucleocytoplasmic shuttling has not been well characterized	[51,57,58,59,60]
Principal biological function	Resolution of inflammation through degradation of cytokine mRNAs	Immune regulation, cellular quiescence, cell-cycle control, genome stability, antiviral defense	Lymphocyte differentiation, cell-cycle control, quiescence and hematopoietic development	Placental RNA regulation	[34,55,56,58,61,62,63]
Major ARE-containing targets	TNF-α, GM-CSF, IL-2, IL-3, IL-6, IL-10, IL-23, CXCL1, CCL2, COX-2 (PTGS2)	Cyclin D3 (Ccnd3), VEGFA, LDLR, Notch1, AU-rich inflammatory transcripts, viral RNAs (virus-dependent)	Cell-cycle regulators (e.g., Cyclin D3), Notch1, lymphocyte developmental transcripts	Placenta-associated AU-rich transcripts (few direct targets identified)	[54,55,56,61,63,64,65]
Regulation of inflammatory cytokines	Strong	Moderate; regulates inflammatory transcripts in a cell type- and stimulus-dependent manner while maintaining immune homeostasis	Indirect; primarily regulates cytokine production through control of lymphocyte development, activation, and differentiation rather, moderately by direct cytokine mRNA decay	Limited evidence	[34,55,56,61,62,63,65]
Autophagy	Limited evidence	Emerging evidence	Not established	Not established	[66,67,68].
Effect on virus infection	Emerging evidence/Proviral (Senecavirus A)	Broad-spectrum antiviral activity	Emerging evidence: antiviral	Not reported	[17,18,19,69,70,71,72,73]
Knockout phenotype	Severe systemic inflammatory syndrome	Embryonic lethality; defects in B-cell development and genome stability	Defective thymopoiesis, impaired hematopoiesis, T-cell abnormalities	No well-characterized knockout phenotype; possible role in extraembryonic development AND placental developmental defects	[53,58,62]

**Table 2:** Lipoprotein receptors exploited by diverse enveloped viruses from different viral families for host cell entry (adapted from François-Loïc et al. [152]).

Virus	Virus Family	Lipoprotein Receptor Used for Entry
Hepatitis C virus (HCV)	*Flaviviridae*	LDL-R, VLDL-R, SR-BI
Dengue virus (DENV)	*Flaviviridae*	LDL-R, SR-BI
Japanese encephalitis virus (JEV)	*Flaviviridae*	LDL-R
Vesicular stomatitis virus (VSV)	*Rhabdoviridae*	LDL-R
Venezuelan equine encephalitis virus (VEEV)	*Togaviridae*	LDLR family receptors
Rift Valley fever virus (RVFV)	*Peribunyaviridae*	LDLR family receptors, LRP1
Sindbis virus (SINV)	*Togaviridae*	LRP8, VLDL-R
Semliki Forest virus (SFV)	*Togaviridae*	LDL-R, LRP8, VLDL-R
Oropouche virus (OROV)	*Peribunyaviridae*	LRP1
Lassa virus (LASV)	*Arenaviridae*	LRP1
SARS-CoV-2	*Coronaviridae*	LDLR family receptors, SR-BI
Hepatitis B virus (HBV)	*Hepadnaviridae*	LDL-R, SR-BI
Classical swine fever virus (CSFV)	*Flaviviridae*	LDL-R
Getah virus (GETV)	*Togaviridae*	LDL-R, LRP8
Ross River virus (RRV)	*Togaviridae*	LDL-R
Western equine encephalitis virus (WEEV)	*Togaviridae*	LDL-R
Eastern equine encephalitis virus (EEEV)	*Togaviridae*	LDL-R

## Data Availability

No new data were created or analyzed in this study. Data sharing is not applicable to this article.
